# Chimpanzees utilize video as reference in a spatiotemporally distant search for hidden food

**DOI:** 10.1098/rsos.240440

**Published:** 2024-09-25

**Authors:** Shenwen Xu, Masaki Tomonaga, Ikuma Adachi

**Affiliations:** ^1^ Center for the Evolutionary Origins of Human Behavior, Kyoto University, Inuyama, Aichi 484-8506, Japan; ^2^ University of Human Environments, Matsuyama, Ehime 790-0825, Japan

**Keywords:** displaced reference, video recognition, video deficit effect, language evolution, chimpanzees

## Abstract

Referring to things that are displaced in space and time is one of the defining features of human language. In order to better understand the evolution of human language, it is therefore important to explore how widely the ability for displaced reference is shared in animal kingdom. In this study, we explored whether chimpanzees are capable of utilizing video as a displaced reference in a spatiotemporally distant task. We used video to inform chimpanzees about an otherwise unobservable food-hiding. We examined the extent to which chimpanzees would make use of video as a source of information to guide their retrieval of hidden food from a target container. We found that when the event of observing food-hiding and the event of retrieving hidden food were close in space and time within the same room, all chimpanzees solved the task. Some chimpanzees continued to solve the task even when the two events were distant and separated spatiotemporally, in which they had to move to the next room between the events. Our findings suggested that chimpanzees can utilize video as a displaced reference to retrieve hidden food later when solving real-life problems.

## 1. Introduction

Referring to things that are displaced in space and time is one of the defining features of human language [1]. Spatiotemporal displacement enormously extends what information a linguistic reference can convey. Referents are thus no longer limited to the things that are within the current perception but also can include the ones that are out of sight [2–5].

Displaced reference is not restricted to language. In nonhuman animal communication systems, a range of suggestive phenomena of the ability for displaced reference have been reported across species (for example, primates act accordingly to vocal calls about out-of-sight predators e.g. [6,7], food [8], third-party interactions [9], travels of sexually mature males [10]; birds act accordingly to calls about out-of-sight predators e.g. [11,12]; honeybees act accordingly to the waggle dances that communicate about out-of-sight food sources e.g. [13]). However, whether and to what extent these displaced references share the underlying cognitive processes with humans remains untested, leaving its evolutionary origins largely unclear [14,15].

To study the ability for displaced reference, video is a validated empirical tool. Seminal studies using video in a hidden-object-retrieval task, revealed the developmental emergence of displaced reference in humans. Children from 2.5 years onwards start to utilize videos that convey information referring to out-of-sight objects and events [16]. They solve the task by making use of video as a source of information later when retrieving the hidden object in the referred room. Given the advantage of the nonlinguistic nature of the task, this approach is promising for empirical exploration in comparative studies with nonhuman animals to better understand the evolution of displaced reference.

Among the animal kingdom, chimpanzees (*Pan troglodytes*) are one of the phylogenetically closest living relatives to humans, making them an ideal species to explore the primate precursors of displaced reference in human language. Moreover, as suggested by previous studies which used videos as stimuli, chimpanzees appear to be a prime candidate species to implement the approach of hidden-object-retrieval task. First of all, chimpanzees show sufficient motivation to visually attend to the videos of realistic depictions of lively events (e.g. [17]). More crucially, they also exhibit behaviours towards the referred objects in real life based on videos (e.g. object search: [18–20]; imitation: [21,22]). These findings raise the possibility that chimpanzees could transfer meaning from video to a real-life experience: namely, that chimpanzees could represent video as a reference to out-of-sight objects and events. However, these studies do not exclude the more parsimonious explanation that chimpanzees’ responses to the referents were solely a result of associative learning over the course of the study [23–25]. In other words, chimpanzees may have simply learned to respond to the objects that had a certain appearance similar to what they saw in the video, resulting from repeated trial and error. Also, these studies do not provide explicit evidence for spatiotemporal displaced reference in chimpanzees because of the procedural nature of the tasks. For example, one line of study demonstrated that chimpanzees utilize live video to guide their hands’ search in an otherwise unobservable space (e.g. [18]). However, the referred objects were within chimpanzees’ sight during the search (i.e. the video served as a spatially displaced reference that was not temporally displaced). Therefore, the task did not essentially require the representation of out-of-sight entities. Another line of study using the object recognition task, showed that chimpanzees utilize recently watched video of a hiding event to search for hidden target (e.g. [19,20]). Nevertheless, the visual features of the target object were continuously perceivable throughout each trial, in which the preceding event of receiving video information and the subsequent event of retrieving objects were within the same space (i.e. the video served as a temporally displaced reference, but it was not spatially displaced). The task thus could be solved simply by using a specific object feature as a cue, rather than by representing video as a reference to out-of-sight entities.

**Figure 1.**
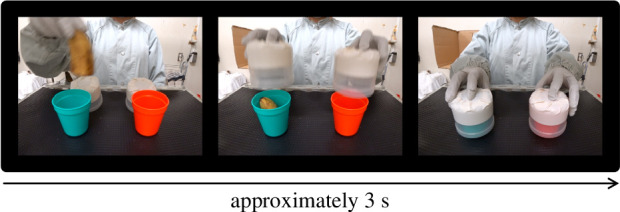
Food-hiding observed by chimpanzees. During the food-hiding, the experimenter first showed the chimpanzee the food item, then placed it in one of the two containers, and then placed lids on both containers.

To address these issues, we tested chimpanzees’ ability to utilize displaced video reference, using the hidden-food-retrieval task modified from previous studies (e.g. [26,27]). In this task, chimpanzees first watched a video of an otherwise unobservable food-hiding (figure 1), in which an experimenter hid a piece of food in one of two opaque containers (observation event). After that, chimpanzees moved to the location of the retrieval task (moving event). They were then offered an opportunity to choose one of the two containers (retrieval event). Through three experiments (see table 1 for an overview), we manipulated the spatial-temporal distance and separation between the observation event and the retrieval event. In experiments 1 and 2, the observation event and the retrieval event were close in time and space, within the same experimental room. The retrieval location was 1 m away, which would take, on average, 3 s to reach from the observation location. Experiments 1 and 2 examined the visual characteristics in the utilization of displaced video reference. In experiment 3, the observation event and the retrieval event were spatially and temporally more distant, across two separate rooms. The retrieval location was 15 m away, which would take, on average, 60 s to reach from the observation location. Experiment 3 examined whether chimpanzees are capable of utilizing the displaced video reference to solve the task, where there is only a minimal opportunity to primarily rely on associating visual features of objects through trial-and-error learning. We predicted that if the chimpanzees were able to utilize the video as a displaced reference, then they would choose the target container with hidden food, even when the observation and retrieval events were separated spatiotemporally.

**Table 1.** Overview of the experimental design.

	experiment 1	experiment 2	experiment 3
spatiotemporal distance between observation event and retrieval event	1 m apart, average 3 s to move; within the same room	1 m apart, average 3 s to move; within the same room	15 m apart, average 60 s to move; across two separate rooms
observation event in each condition	real condition: observe directly or through live video	real condition: observe directly or through live video	real condition: observe directly
	video condition: observe through live video	video condition: observe through live video	video condition: observe through live video
testing order	real condition → video condition	real condition → video condition	video condition → real condition
number of trials for each session	12 trials per session	12 trials per session	—
number of sessions for each container pair	6 sessions per pair	3 sessions per pair	—
number of container pairs	1 pair	3 pairs	1 pair
number of trials for each condition	72 trials per condition	108 trials per condition	16 trials per condition

## 2. Experiment 1

In experiment 1, we examined whether chimpanzees can retrieve hidden food immediately after they observed a food-hiding through video. The observation location and the retrieval location were 1 m apart. It took 3 s, on average, for the chimpanzee to move from the observation location to the retrieval location. On each trial, a piece of food was hidden in either one of the two opaque containers.

### 2.1. Methods

#### 2.1.1. Participants

We tested seven chimpanzees (one male and six females, ranging from 18 to 43 years old, see table 2 for more detailed information), who were socially housed at the Primate Research Institute, Kyoto University (see [30] for details). They participated in the experiments voluntarily. They could choose to come to the experimental room and to stop participating at any point. They had access to the experimental room through pathways, connected to indoor and outdoor enclosures (700 m^2^), which were furnished with over 60 species of plants, three-dimensional climbing structures, and various enrichment feeders [31]. They were never food-deprived. They were fed three to six times a day with a variety of fruits and vegetables, and primate biscuits. Water was available ad libitum. The study was carried out between May 2019 and February 2020. The study adhered to the Guidelines for Care and Use of Nonhuman Primates of the Primate Research Institute, Kyoto University (version 3). The study protocol was approved by the Animal Research Committee and the Animal Welfare and Animal Care Committee of the Primate Research Institute, Kyoto University (reference number: 2019-192, 2020-211).

**Table 2.** Information on the chimpanzees that participated in this study.

participant (GAIN^a^ ID)	sex	age^b^	participation
Ai (0434)	female	43	experiments 1–3
Ayumu (0608)	male	19	experiments 1–3
Cleo (0609)	female	18	experiments 1–3
Chloe (0441)	female	38	experiments 1–3
Pal (0611)	female	18	experiments 1–3
Pan (0440)	female	35	dropped out during the real condition of experiment 1
Pendesa (0095)	female	42	dropped out during the real condition of experiment 1

^a^ Great Ape Information Network (GAIN) is an open-access nationwide database containing the detailed life-history information of all apes who have lived or currently reside in Japan (https://shigen.nig.ac.jp/gain/index.jsp; see [28,29]).

^b^ As of May 2019, the beginning of this study.

Several earlier experiences may be relevant to the performance of the chimpanzees in the current experiment. In terms of their exposure to live videos, some of the chimpanzees (Chloe, Ai, Pan, Pendesa) had been tested on a self-image recognition task, where they had a chance to explore the temporal contingency between the movement of their own body and its live video image [32]. However, they were not involved in any tasks where they needed to utilize the video image of currently unobservable objects and events. Regarding the procedure of object choice tasks, all seven chimpanzees had extensive experience choosing targets on touchscreens, including static images that retain some similarity to the actual entities (e.g. photographs) and the ones that are abstracted away from the actual entities (e.g. lexigram, Arabic numeral; e.g. [33,34]). All the chimpanzees also had some experience choosing between real objects (e.g. [35–37]). However, both the given cues and choices were always within the same dimension. Therefore, no one had been exposed to any tasks where they were required to utilize information in the video to make a choice towards the objects in front of them.

#### 2.1.2. Set-up and procedure

**Figure 2.**
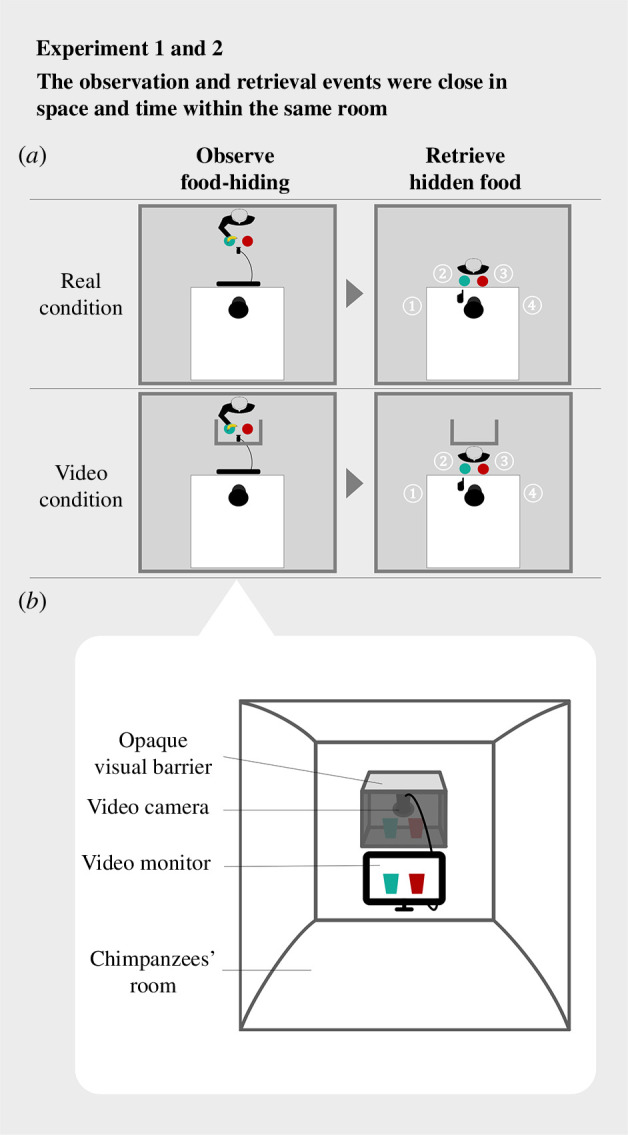
Sketch of the hidden-food-retrieval task used across experiments 1 and 2. Throughout each trial, the observation and retrieval events were close in space and time within the same room. (*a*) Timeline of the events on each trial from a top view. During the observation event, the chimpanzee could observe the food-hiding directly as well as through video in the real condition, but only through video in the video condition. Immediately after the observation event, the chimpanzee could directly see the two containers which were moved to the retrieval location by the experimenter. The chimpanzee followed the experimenter to the retrieval location and then chose one of the containers. One of four possible retrieval locations, denoted by the white letters 1–4, was randomly used on each trial. This ensured that the chimpanzee could not use any alternative memory strategies, such as orienting towards the corresponding side of the target container (left or right) during the observation event. Upon a correct choice to the target container, the chimpanzee was rewarded with the food. (*b*) The set-up used in the video condition. The video monitor was connected to the video camera. Food-hiding video was captured by the camera and was shown on the monitor in real time.

Chimpanzees participated in all experiments individually. They received the experiments in a room (1.8 × 1.8 × 2.1 m^3^, figure 2) covered with transparent acrylic panels. From inside their room, the apparatuses were readily visible but out of reach. There were two conditions: real condition, in which chimpanzees could observe the food-hiding either directly or through a live video, and video condition, in which they could observe the food-hiding only through a live video. The two conditions had the same general set-up and procedure. On each trial, three events occurred one after another: observation, moving, and retrieval (figure 2*a*).

##### 2.1.2.1. Observation event

During the observation event, chimpanzees observed the food-hiding. Before the food-hiding started, an experimenter (S.X.) presented food (a half piece of peanut) to the chimpanzee in order to attract their attention. Chimpanzees were guided to sit at the observation location in front of two opaque containers. The containers (a green box and a red cup) served as a target and a distractor container (table 3). A combination of three types of visual feature of the containers (colour: green and red; shape: trapezium and rectangle; side: left and right) were available to distinguish between the containers. The containers were placed side by side on a transparent platform 100 cm off the floor, ensuring that chimpanzees could not see the inside of the containers. Below the platform, a 23-inch LED monitor (IPS235VX, LG Electronics, Japan) for video presentation was placed 40 cm off the floor, so that the centre of the monitor was approximately at the eye level of a sitting chimpanzee. The video of food-hiding was captured using a video camera (Action Cam FDR-X3000, Sony, Japan) and was shown on the monitor in real time. The video depicted the food, both containers, the experimenter’s hands, and the full motion trajectory of the food being hidden in a container by the experimenter (figure 1). The size, colour, and angle of the video depicted scene was comparable to the one seen directly from the front of the containers from inside the chimpanzees’ room. While chimpanzees could see the food-hiding directly as well as through video in the real condition, in the video condition we added an opaque box-shaped screen to block the chimpanzees from directly observing the food-hiding and thus they could only see it through video (figure 2*b*). The screen was made of a cardboard box (45 × 47 × 55 cm^3^), placed on the platform. It was closed on five sides and opened on one side for the experimenter to hide the food inside the box. One of the five closed sides, which was in contact with the platform, had two peepholes (diameter 6 cm) through which the experimenter could check if the chimpanzee was visually attending to the video before start the food-hiding. Each trial was initiated as soon as the chimpanzee visually attended to the food. If the chimpanzee was not visually attending to the food, the experimenter slightly swung their hand with the food above the containers or called the chimpanzee’s name to attract their attention. The food was then hidden in one of the two containers by the experimenter.

**Table 3.** Overview of the container pairs used in each experiment**.** (On each trial, food was being hidden in either one of the opaque containers. The container with hidden food served as the target and the other container was a distractor.)

	experiment 1	experiment 2			experiment 3
available feature(s)	side + shape + colour	side	shape	colour	colour
container pair	green box, red cup	white cup, white cup	green box, green cup	green cup, red cup	green cup, red cup
	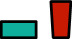	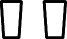	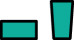	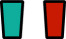	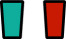

##### 2.1.2.2. Moving and retrieval event

Immediately after the observation event, the two containers were moved to the retrieval location by the experimenter, and directly presented to the chimpanzees. Chimpanzees followed the experimenter to the retrieval location and then chose one of the containers by pointing (i.e. extending a hand or finger towards it). At the retrieval event, the relative side of the two containers was the same as those in the observation event. There were four retrieval locations (on the left or diagonally left or diagonally right or right of the observation location) (figure 2*a*). On each trial, one of them was randomly used. This design was to ensure that the chimpanzees could not use any alternative memory strategies, such as orienting towards the corresponding side of the target container (left or right) during the observation event. If the chimpanzees chose the target container (correct choice) the experimenter handed the food as a reward to them through a feeding tube. If they chose the distractor container (incorrect choice) the experimenter showed them the inside of both containers and withdrew the food.

Each chimpanzee received one session per day. Each session consisted of 12 trials: three repetitions of the four possible combinations of the two containers (a green box and a red cup) and two sides (left or right). The 12 trials were presented in pseudorandom order, ensuring that the target was not assigned to the same container on the same side for more than two successive trials. Chimpanzees whose performance was significantly better than expected by chance across six sessions (41 correct trials out of 72 or better, *p* < 0.05, two-tailed binomial test) of the real condition proceeded to the video condition (table 1). The video condition was conducted for six sessions.

### 2.1.3. Analysis

Two out of seven chimpanzees (Pendesa and Pan) dropped out at the beginning of the real condition because they were not motivated to pay enough attention to the food-hiding. The other five chimpanzees received both the real and video conditions. Therefore, the analysis included the performance of these five chimpanzees in both conditions. The performance of each trial was coded either as 1 when it was correct or as 0 when it was incorrect.

To evaluate the performance at group level, we ran binomial generalized linear mixed models (GLMMs) with logit link function and with significance level alpha at 0.05. We used the glmer function in the lme4 package [38] with R [39] and RStudio (version 2023.9.1.494) [40].

To determine whether chimpanzees utilized information from the observation event in the retrieval task, we compared their proportion correct to 50% chance. For each condition, we fitted an intercept-only model (formula: correct ~ 1 + (1 | ID)) that predicts 50% chance and tested whether the estimated intercept differed from zero.

To calculate which of the factors had significant effects on correct performance, we selected a model that was the best fit for the total dataset across both conditions, based on the Akaike Information Criterion (AIC). Specifically, we first included the fixed effects of condition (real, video), session number (1–6) and their two-way interactions and a random effect of chimpanzee identity (ID) in the initial model. Then, a set of models with all possible combinations of the fixed effects of the initial model was generated and AICs of the models were compared, using the dredge function in the MuMIn package [41]. The significance of individual factors was obtained by comparing between the best fitting model (with the factor) and the respective reduced model (without the factor) using likelihood ratio tests with the anova function. Since only the factor of condition significantly affected the performance, we then used the same model to compare the performance between conditions. We applied Tukey’s test for pairwise comparisons between conditions within the best fitting model of both conditions, using the glht function in the multcomp package [42].

### 2.2. Results

**Figure 3.**
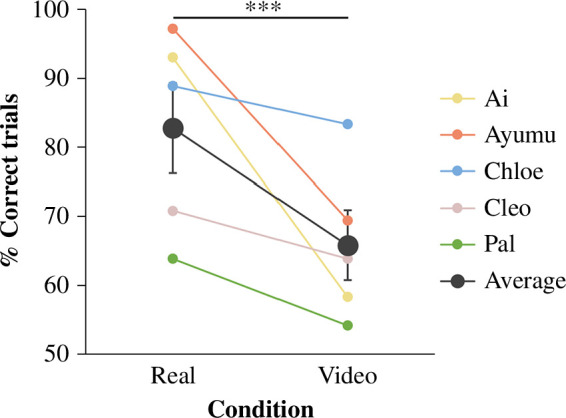
Performance of the real condition and the video condition in experiment 1. Percentage of correct trials differed between conditions. Black dots represent group mean. Error bars represent standard errors of the group mean. Coloured dots represent individual mean. In each condition, there were a total of 72 trials per chimpanzee. ***p < 0.001 (GLMM).

On average, of the five chimpanzees, the performance was above chance expectation in both conditions (real condition: estimate = 1.83, *p* < 0.001, 95% confidence interval (CI) [0.64, 3.15]; video condition: estimate = 0.68, *p* = 0.00153, 95% CI [0.17, 1.22]; figure 3). The best fitting model for the data across both conditions contained only the fixed effect of condition. The model fit was significantly improved with the inclusion of the factor of condition ($\chi_{1}^{2}$ = 6.45, *p* < 0.001), while this was not the case for the factor of session number ($\chi_{1}^{2}$ = 1.61, *p* = 0.2038), nor the interaction between session number and condition ($\chi_{1}^{2}$ = 2.03, *p* = 0.3624). Therefore, only condition significantly affected the performance. Compared to the real condition, performance in the video condition was significantly lower (GLMM, Tukey’s test: *z* = −4.89, *p* < 0.001; figure 3).

### 2.3. Discussion

Experiment 1 confirmed that chimpanzees are capable of retrieving hidden food by utilizing information from video watched 3 s earlier. However, the results also revealed a declined performance in the video condition compared with that of the real condition, suggesting that the perceptual experiences of each trial between the conditions (video condition: seeing the target object in video at the observation location and then seeing it in real life at the retrieval location, real condition: seeing the target object in real life at the observation location and then seeing it in real life at the retrieval location) were not equivalent. The findings are in line with previous studies both in human children (e.g. [43–45], see [46] for a review) and in nonhuman animals (e.g. capuchin monkeys (*Sapajus apella*): [47], California scrub-jays (*Aphelocoma californica*): [48]). This phenomenon is known as ‘video deficit effect’, whereby an inferior task performance can occur when the real-life problem needs to be solved based on information received from video compared with from real-life experience. To further evaluate the visual-related effects of video deficit in the current study, we thus decomposed the visual features of objects into more primitive types and scrutinized their effectiveness in experiment 2.

## 3. Experiment 2

Given that chimpanzees could utilize video information to retrieve hidden food when a combination of visual features between the two containers was available, we then introduced experiment 2 to determine more specifically how much each feature was relied on. We broke down the featural components (the relative side, shape, and colour of the container), that were used in experiment 1, and examined them independently.

### 3.1. Methods

#### 3.1.1. Participants

The same five chimpanzees who performed significantly well in experiment 1 (see table 2).

#### 3.1.2. Set-up and procedure

The set-up was mostly the same as that used in experiment 1, except that in experiment 2, there were a total of three pairs of opaque containers, each pair employed one type of visually distinguishable feature. The two white cups were different in relative left or right side (side pair), a green trapezium cup and a green rectangle box were different in shape (shape pair), and a green cup and a red cup were different in colour (colour pair) (for illustrations of each container pair, see table 3).

The procedure of each trial was similar to that in experiment 1, except for one additional manipulation in order to make the feature of containers’ relative side (left and right) unusable when distinguishing the shape and colour pairs. Specifically, the containers’ relative side had been switched at the retrieval event in four random trials out of 12 within each session of the shape and colour pairs. They were switched by the experimenter after presenting the food-hiding to the chimpanzees and before moving to the retrieval location, while the containers were briefly obscured from the chimpanzees’ view.

As in experiment 1, each session in experiment 2 also consisted of 12 trials. All the sessions of the real condition were conducted prior to the video condition (table 1). Each of the three container pair types was repeated for three sessions in each condition, yielding a total of nine sessions in the real condition and nine sessions in the video condition. Each chimpanzee received one session per day. The presentation order of the pair types was counterbalanced within and across chimpanzees.

#### 3.1.3. Analysis

The statistical analysis of experiment 2 followed the same general procedure as experiment 1.

To determine whether chimpanzees utilized information from the observation event in the retrieval task, we compared their proportion correct to 50% chance. For each condition, we fitted an intercept-only model (formula: correct ~ 1 + (1 | ID)) that predicts 50% chance and tested whether the estimated intercept differed from zero.

To examine which of the factors had an effect on the performance across conditions and to compare the performance between conditions, we selected the best fitting model for the total dataset across both conditions. We included condition (real, video), pair type (side, shape, colour), session number (1–3) and their two-way interactions as the fixed effects, and chimpanzee ID as the random effect in the initial model. Since the best fitting model revealed a significant difference between the real and video conditions, the two conditions were thereafter analysed separately. We applied Tukey’s test for pairwise comparison between the pair types within the GLMM of each condition, using the glht function in the multcomp package [42].

### 3.2. Results

**Figure 4.**
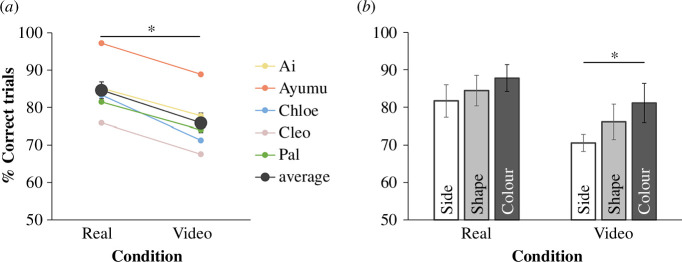
Performance on the real condition and the video condition in experiment 2. (*a*) Across the three types of container pair, percentage of correct trials differed between the real and video condition. Black dots represent group mean. Coloured dots represent individual mean. (*b*) Within each condition, percentage of correct trials of each type of the container pair was compared. Significant difference was found between the pair type of side and colour in the video condition, but not between other pair types. Bars represent group mean. For both (*a*) and (*b*), error bars represent standard errors of the group mean. In each condition, there were a total of 108 trials per chimpanzee. *p < 0.05 (GLMM).

The average performance of the five chimpanzees was above chance expectation in both conditions across the three container pair types (real condition: estimate = 1.73, *p* < 0.001, 95% CI [0.77, 2.79]; video condition: estimate = 1.18, *p* < 0.001, 95% CI [0.72, 1.68]; figure 4*a*). The performance of each type of the container pair was also above chance expectation in both the real condition (side pair: estimate = 1.45, *p* < 0.001, 95% CI [0.61, 2.40]; shape pair: estimate = 1.71, *p* < 0.001, 95% CI [0.63, 3.28]; colour pair: estimate = 1.98, *p* < 0.001, 95% CI [0.98, 3.48]) and the video condition (side pair: estimate = 0.87, *p* < 0.001, 95% CI [0.56, 1.21]; shape pair: estimate = 1.20, *p* < 0.001, 95% CI [0.61, 1.93]; colour pair: estimate = 1.55, *p* < 0.001, 95% CI [0.82, 2.43]) (figure 4*b*).

The best fitting model for the data across both conditions contained the fixed effects of condition, pair type, and session number. Condition ($\chi_{1}^{2}$ = 6.45, *p* = 0.011) and pair type ($\chi_{2}^{2}$ = 8.56, *p* = 0.014) significantly affected on the improvement of model fit, whereas session number ($\chi_{1}^{2}$ = 3.56, *p* = 0.059) did not. Chimpanzees performed significantly worse in the video condition compared with the real condition (estimate = −0.39, *p* = 0.011, 95% CI [-0.70, -0.09]; figure 4*a*). Within the real condition, there was no significant difference between the pair types (GLMM, Tukey’s test: side-colour pair comparison, *z* = −1.722, *p* = 0.197; shape-colour pair comparison, *z* = −1.038, *p* = 0.552; shape-side pair comparison, *z* = 0.695, *p* = 0.766; figure 4*b*). Within the video condition, the chimpanzees’ performance was significantly worse in the side pair than the colour pair (GLMM, Tukey’s test: side-colour pair comparison, *z* = −2.353, *p* = 0.0487), while no significant difference was found between the other pair types (GLMM, Tukey’s test: shape-colour pair comparison, *z* = −1.168, *p* = 0.4718; shape-side pair comparison, *z* = 1.206, *p* = 0.4493) (figure 4*b*).

### 3.3. Discussion

Across the three types of container pair, chimpanzees performed above chance expectation in all of the video conditions. This suggested that when there was only one type of visually distinct feature between the target and distractor container, chimpanzees could still utilize video information to locate the target container.

Consistent with experiment 1, performance in the video condition was worse than in the real condition, showing a continued video deficit effect. Notably, in the video condition, performance of the side pair was significantly worse than the colour pair. Such impaired effectiveness of the side feature as a cue for object identification may be attributed to the spatiotemporal displacement of the containers between the events of receiving and utilizing video information. That is, accompanied by the displacement, the relative side feature between the containers is not as invariant compared with the colour feature. Additionally, the video deficit effect might result in a vision deficit in processing the video image during the observation event. Specifically, the visual system that determines where objects are when organizing a scene in three dimensions, might be weakened when organizing a scene in two dimensions [49]. As a result, the object recognition and the location recognition had different importance when identifying the corresponding objects later in real life. Another alternative account might be related to chimpanzees’ earlier experience. All chimpanzees have had extensive experience with matching-to-sample tasks on touchscreen, which mostly require attention towards features of the objects themselves rather than their relations, such as relative side. To examine how much this object feature matching experience contributed to the current result, the same test with a different population of naive chimpanzees will provide more compelling evidence.

Experiments 1 and 2 confirmed the basic ability for displaced video reference. This set the base for a more challenging version of the task in experiment 3, embedded with a more distant spatiotemporal separation between events as well as a minimal opportunity to solve the task based primarily on associating visual features of objects through trial-and-error learning.

## 4. Experiment 3

In experiment 3, the observation event and the retrieval event were spatiotemporally distant and separated. Between the events, chimpanzees had to move across two rooms. The retrieval location was 15 m away, which would take, on average, 60 s to reach from the observation location. We examined whether chimpanzees would still utilize video as a displaced reference later when retrieving a hidden food.

### 4.1. Methods

#### 4.1.1. Participants

The same five chimpanzees who performed significantly well in experiments 1 and 2 (see table 2).

#### 4.1.2. Set-up and procedure

**Figure 5.**
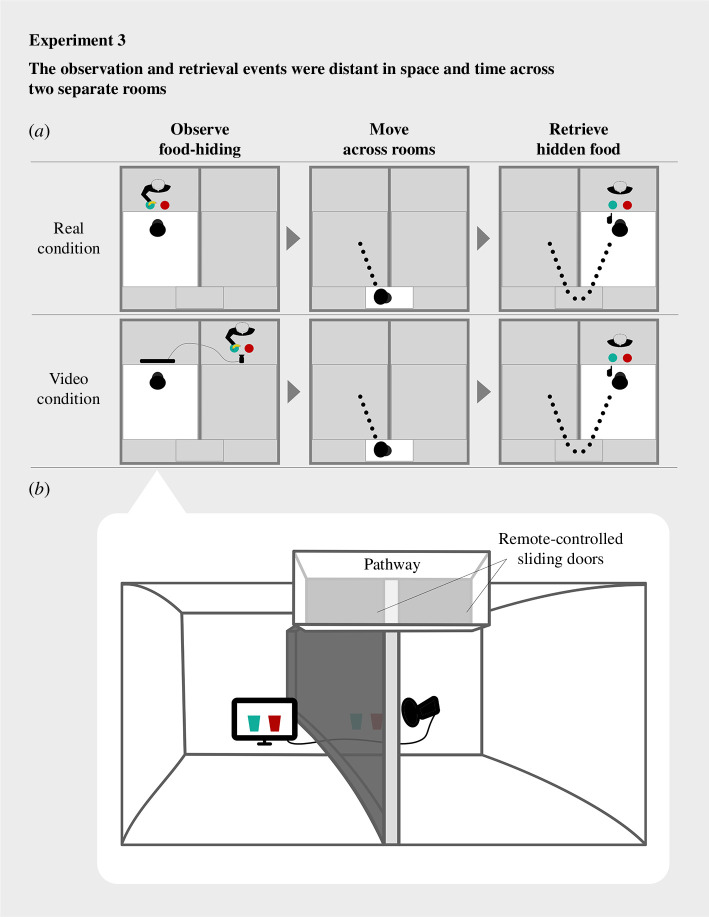
Sketch of the hidden-food-retrieval task used in experiment 3. Throughout each trial, the observation and retrieval events were distant and separated spatiotemporally across rooms. (*a*) Timeline of the events on each trial from a top view. During the observation event, the chimpanzee observed food-hiding directly in the real condition, and through video in the video condition. Then, the chimpanzee moved from the observation room to the pathway and into the retrieval room. The doors of the rooms first were opened for chimpanzees to move from the observation room to the pathway and into the retrieval room, then were closed so chimpanzees could not back track. The moving took approximately 60 s. At the retrieval event, the chimpanzee chose one of the containers. Upon a correct choice to the target container, the chimpanzee was rewarded with the food. (*b*) The apparatuses and two rooms used in the video condition. The video monitor was connected to the video camera across the rooms. The food-hiding video was captured by the camera and was shown on the monitor in real time.

Experiment 3 was conducted after the completion of experiment 2 (the time gap was between 3 and 12 days, depending on whether it was available to recruit the individual chimpanzee). Unlike in experiments 1 and 2, throughout each trial, chimpanzees moved across two adjacent rooms (figure 5). Each room was visited once in a given sequence (figure 5*a*). In the first room (hereafter ‘observation room’), chimpanzees observed the food-hiding. In the second room (hereafter ‘retrieval room’), they could get the food if they chose the target container.

It is important to note that the testing schedule of experiment 3 was designed to more explicitly rule out the possibility of learning effects across the trials of both the video and real conditions (see §4.1.2.1 below for details). Each condition consisted of 16 trials in total, which was a minimum number to statistically determine whether each chimpanzee’s performance differed from what would be expected by random chance. In addition, all the trials of the video condition were presented prior to the real condition (table 1). This was done for maximizing the novelty of the displaced video reference in the context where the observation and retrieval events were separated spatiotemporally across rooms. Each chimpanzee participated in one trial per day. Within each condition, each of the four possible combinations of the two containers (a green cup and a red cup) and two sides (left or right) was repeated four times. They were presented in pseudorandomized order across trials.

##### 4.1.2.1. Observation event

Across both the video and real condition, the food-hiding was shown from the point of view of a sitting chimpanzee observing from diagonally above the containers (figure 1). The insides of the opaque containers (a green cup and a red cup) were not observable once they were covered by the opaque lids. We added the lids in experiment 3 to ensure that while moving across rooms, during which time the chimpanzees sometimes climbed up to higher places, they still could not see the inside of the containers. To give chimpanzees enough motivation in experiment 3, since each trial demanded more effort (e.g. cross-room moving, delayed reward) than in experiments 1 and 2, we used a half banana (more desirable than a half piece of peanut, that was used in experiments 1 and 2) in the food-hiding and as the reward upon a correct choice. The food-hiding was initiated once the chimpanzees started visually attending to the food. The banana was first hidden in one of the two cups, and then both cups were covered by lids simultaneously. The food-hiding lasted for approximately 3 s.

In the video condition, the food-hiding was shown to the chimpanzees through a live video in the observation room (figure 5*b*). It was demonstrated by experimenter A (S.X.) in the retrieval room, towards the camera that connected to the video shown in the observation room. After hiding the food, experimenter A held the cups one in each hand and moved out from the field of the video with the cups. Chimpanzees could not see the cups from then on. Meanwhile, before the food-hiding start, experimenter B (one of the laboratory assistants in charge of the day, E.I. or T.T., see Acknowledgements) stayed behind the video monitor in the observation room, and checked whether the chimpanzee’s visual attention was directed to the video. In case the chimpanzee was not looking towards the video, experimenter B called the chimpanzee’s name to attract their attention.

In the real condition, the food-hiding was shown directly in the observation room. It was demonstrated by experimenter A in front of the chimpanzee, in the same manner as that in the video condition. After hiding the food, experimenter A held the cups one in each hand and walked out of the observation room, therefore out of chimpanzees’ sight.

##### 4.1.2.2. Moving event

Once the food was hidden, chimpanzees moved from the observation room to the retrieval room. The two adjacent rooms were identical in size (each 1.8 × 1.8 × 2.1 m^3^; figure 5). The rooms were divided by a concrete wall and were externally connected by a pathway (at 2.1 m height, 7 m long) that was attached to the outside of the door of each room. When chimpanzees entered or left the room, the doors on either side of the pathway were remotely controlled by experimenter B, which was a familiar manipulation to the chimpanzees prior to this study. Before the moving, the door of the observation room was kept closed during the chimpanzee’s observation of the food-hiding. Once the observation event was completed, experimenter B opened the door of the observation room and the chimpanzee would move out from the observation room to the pathway. Experimenter B then closed the door of the observation room so the chimpanzee could not back track, and then opened the door of the retrieval room for the chimpanzee to move in, and closed it after the chimpanzee entered the room. The whole moving process across the two rooms (i.e. climbing up and down the ladders below each door and walking through the pathway) took approximately 60 s.

##### 4.1.2.3. Retrieval event

Once the chimpanzee arrived in the retrieval room, they saw experimenter A with the two cups. The relative side of the two containers were the same as those in the observation event. Chimpanzees made their choice between the cups by pointing. After that, experimenter A removed the lids of both cups simultaneously and showed the inside of the cups to the chimpanzee. Following a correct choice, experimenter A handed the food to the chimpanzee through a feeding tube. Following an incorrect choice, experimenter A showed the chimpanzee the inside of both containers and withdrew the food.

### 4.1.3. Analysis

The performance was analysed at individual level using one-tailed binomial tests with the significance level alpha at 0.05. We assessed whether the chimpanzees performed differently than expected by 50% chance (i.e. correct trials numbered above 12 or below 4 out of 16).

We also examined whether the performance of each chimpanzee increased over the course of the 16 trials within each condition using Pearson’s correlation tests.

### 4.2. Results

**Figure 6.**
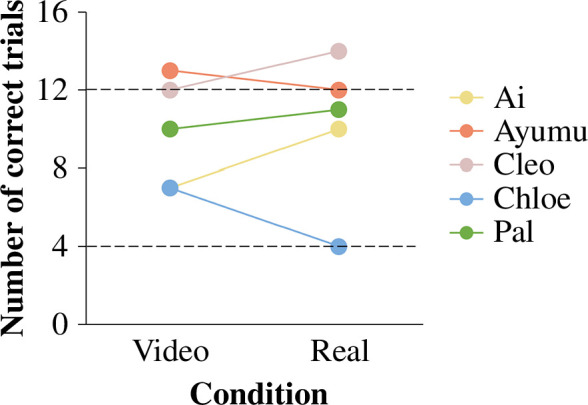
Number of correct trials of the video condition and the real condition in experiment 3. Coloured dots represent individual performance. In each condition, there were a total of 16 trials per chimpanzee. Dashed lines denote significance level above or below random chance expectation: 12 or 4 trials (*p* < 0.05, binomial test).

Two chimpanzees performed better than expected by chance both in the video condition (Ayumu: 13 correct trials, binomial test *p* = 0.01064; Cleo: 12 correct trials, binomial test *p* = 0.03841) and in the real condition (Ayumu: 12 correct trials, binomial test *p* = 0.03841; Cleo: 14 correct trials, binomial test *p* = 0.00209) (figure 6). Two chimpanzees’ performance did not differ significantly from chance expectation either in the video condition (Ai: seven correct trials, binomial test *p* = 0.7728; Pal: 10 correct trials, binomial test *p* = 0.2272) or in the real condition (Ai: 10 correct trials, binomial test *p* = 0.2272; Pal: 11 correct trials, binomial test *p* = 0.1051) (figure 6). One chimpanzee’s number of correct trials did not differ significantly from chance expectation in the video condition (Chloe: seven correct trials, binomial test *p* = 0.7728) and was below chance expectation in the real condition (Chloe: four correct trials, binomial test *p* = 0.03841) (figure 6).

In the video condition, there was no significant correlation between trial number and performance within the 16 trials, in any of the five chimpanzees (Ayumu: *r*_14_ = 0.08684168, *p* = 0.7491; Cleo: *r*_14_ = −0.1252449, *p* = 0.644; Ai: *r*_14_ = −0.06832667, *p* = 0.8015; Pal: *r*_14_ = 0.3640728, *p* = 0.1657; Chloe: *r*_14_ = 0.040996, *p* = 0.8802). In the real condition, trial number and performance within the 16 trials was found to be moderately correlated in one chimpanzee (Ai: *r*_14_ = 0.5041008, *p* = 0.04647), but not in other chimpanzees (Ayumu: *r*_14_ = 0.438357, *p* = 0.08943; Cleo: *r*_14_ = 0.20498, *p* = 0.4463; Pal: *r*_14_ = −0.424138, *p* = 0.1016; Chloe: *r*_14_ = −0.4696682, *p* = 0.06642).

### 4.3. Discussion

Two out of five chimpanzees solved the tasks in the video condition. This suggested that when the observation and retrieval events were separated spatially and temporally into two rooms, some chimpanzees could still utilize video as a displaced reference to specify the target container. We found no evidence for learning effects in the two chimpanzees, confirming that their performance was not merely a result of trial-and-error learning over the course of the 16 trials in any condition. Notably, only these two chimpanzees who solved the earlier tasks in the video condition were able to solve the latter tasks in the real condition, indicating that the processes required for these tasks involved not only the similarity relation between the objects in video and in real life, but also the spatial-temporal contiguity relation between the events across the rooms [27].

Such aggregate demands could have led to insufficient motivation to solve the task, as reflected by chimpanzee Chloe’s performance in the real condition, which was significantly lower than expected by random chance. Also, it could have accounted for a gradual shift of strategy to rely primarily on simpler associative rules in solving the task, as shown by the learning effect of chimpanzee Ai in the real condition, which was not the case in the preceding trials of the video condition.

## 5. General discussion

The current study examined chimpanzees’ ability to utilize video as a displaced reference when retrieving hidden food. After chimpanzees observed the food-hiding through video, they were offered an opportunity to retrieve the hidden food by identifying the target container. Overall, the results illustrated that when the events of observation and retrieval were close in time and space within the same room, all five chimpanzees were able to solve the task (experiments 1 and 2). When the two events were spatiotemporally separated across rooms, two out of five chimpanzees were still able to solve the task (experiment 3).

‘Dual representation’ has been proposed to explain the cognitive challenge in utilizing displaced references to specify the out-of-sight target entities. That is, one must hold in mind reference X’ and referent X based on both (i) the relation of a concrete/visible form and (ii) the relation of an abstract/indirect rule [24,27,50–53]. More specifically, in the context of the current task, at the time of the choice between the target and distractor container, the chimpanzee needed to: (i) relate the target container that was previously observed through video to the target container that was currently present in real life based on featural similarity (a relation of concrete/visible form), and (ii) relate the previous observation event to the current retrieval event based on spatial-temporal contiguity (a relation of abstract/indirect rule). Our results therefore supported the notion that displaced video reference cannot be achieved by simpler associative strategies when solving tasks as in the current study. It is noteworthy that only the chimpanzees who solved the preceding video condition solved the subsequent real condition in experiment 3. This provided further support that between the visual experience of the observation event and the retrieval event, not only featural similarity of the objects but also spatial-temporal contiguity of the events need to be integrated to solve the tasks of both conditions in experiment 3. Additionally, we found no learning effect across the video conditions in any of the three experiments, ruling out the possibility that the ability to utilize the displaced video reference was only a result of trial-and-error learning from repeated exposure to the task. Taken together, these findings expanded on which cognitive faculties are involved when chimpanzees make use of video as a displaced reference. Future research will be important to examine more specifically how such dual processing jointly maintains and manipulates both the concrete items and the abstract rules during the utilization of displaced reference.

It remains unanswered whether the chimpanzees who were not able to integrate the spatial-temporal contiguity of the events was simply owing to a lack of sufficient experience to expect a set of events across different rooms to be as relevant as one whole task. Such a possibility should be examined in future research by increasing the perceivable task continuity (e.g. request for immediate response soon after chimpanzees watched the video before they leave the room). Also, it remains possible that the statistically nonsignificant performance in experiment 3 may not necessarily reflect the inability of these chimpanzees to solve the task since the significance was determined based on a small number of trials and they were obtained only at a single time per day. Further tests with a different testing schedule, that is less susceptible to the potential fluctuations in each day and each individual, may reveal their ability in this task. Moreover, it remains an open question whether differences in the performance of utilizing displaced video reference depend on the patterns of visual information processing, given the individual differences found in this study. Using eye tracker with the established technique, that was tailored for examining chimpanzees’ gaze behaviour during video presentation (e.g. [54,55]), can be of help to elucidate such attentional mechanisms in a finer temporal resolution.

In conclusion, the current study showed that chimpanzees can utilize video as a reference to observe out-of-sight events and localize objects when solving real-life problems, in a manner analogous to a rudimentary form of the displaced reference in human language. The underlying cognitive processes of displaced reference, which are shared between chimpanzees and humans, are thus not necessarily shaped by a fully-fledged linguistic competence. To further research the ability for spatiotemporal displaced video reference in nonhumans, as demonstrated here in this study, the hidden-object-retrieval task can be a feasible approach.

## Data Availability

The datasets and R scripts are publicly accessible on the Figshare repository: [56].
